# Sensitivity of plant species to warming and altered precipitation dominates the community productivity in a semiarid grassland on the Loess Plateau

**DOI:** 10.1002/ece3.5312

**Published:** 2019-06-13

**Authors:** Fanglong Su, Yanan Wei, Fuwei Wang, Jiuxin Guo, Juanjuan Zhang, Yi Wang, Hui Guo, Shuijin Hu

**Affiliations:** ^1^ Ecosystem Ecology Lab, College of Resources and Environmental Sciences Nanjing Agricultural University Nanjing China; ^2^ International Magnesium Institute, College of Resources and Environment Fujian Agriculture and Forestry University Fuzhou City China; ^3^ State Key Laboratory of Loess and Quaternary, Institute of Earth Environment Chinese Academy of Sciences Xi'an China; ^4^ Department of Entomology & Plant Pathology North Carolina State University Raleigh North Carolina

**Keywords:** aboveground net primary productivity, plant community, plant interspecific relationship, soil moisture, tolerance to drought

## Abstract

Global warming and changes in precipitation patterns can critically influence the structure and productivity of terrestrial ecosystems. However, the underlying mechanisms are not fully understood. We conducted two independent but complementary experiments (one with warming and precipitation manipulation (+ or – 30%) and another with selective plant removal) in a semiarid grassland on the Loess Plateau, northwestern China, to assess how warming and altered precipitation affect plant community. Our results showed that warming and altered precipitation affected community aboveground net primary productivity (ANPP) through impacting soil moisture. Results of the removal experiment showed competitive relationships among dominant grasses, the dominant subshrub and nondominant species, which played a more important role than soil moisture in the response of plant community to warming and altered precipitation. Precipitation addition intensified the competition but primarily benefited the dominant subshrub. Warming and precipitation reduction enhanced water stresses but increased ANPP of the dominant subshrub and grasses, indicating that plant tolerance to drought critically meditated the community responses. These findings suggest that specie competitivity for water resources as well as tolerance to environmental stresses may dominate the responses of plant communities on the Loess Plateaus to future climate change factors.

## 1 INTRODUCTION

Global warming and increasing variability in precipitation are important components of the ongoing climate change (IPCC, 2013). The average earth surface temperature has increased by 0.85°C from 1880 to 2012 and is expected to continue to rise in the 21st century (IPCC, 2013). Besides, the average precipitation of the Northern Hemisphere tended to increase since 1901 (IPCC, 2013). Moreover, climate warming will likely increase extreme precipitation events (Allan & Soden, 2008; Goswami, Venugopal, Sengupta, Madhusoodanan, & Xavier, 2006). Therefore, warming and changes in precipitation may interactively affect plant communities and their productivity.

Warming can affect plant growth both positively and negatively. On one hand, warming may enhance plant growth through altering plant physiologies and nutrient availability. Warming can directly change plant photosynthesis (Klanderud & Totland, 2005) and thus alter plant growth rate (Walther & Burga, 2005). In a warmer climate, plants germinate earlier in spring and senescence later in autumn, increasing the length of the growing season (Sullivan & Welker, 2005; Xu, Hu, & Zhang, 2012). Also, warming can stimulate soil nitrogen mineralization to provide more nutrients for plants to grow (Melillo et al., 2002). On the other hand, warming may suppress plant growth by aggravating water stress, particularly in arid and semiarid regions via increasing ecosystem evapotranspiration (Bai, Han, Wu, Chen, & Li, 2004) and decreasing soil water availability (Niu et al., 2008; Wan, Xia, Liu, & Niu, 2009). In semiarid regions, water is the most important factor affecting plant growth (Haase, Pugnaire, Clark, & Incoll, 1999; Raich et al., 1991). While increased precipitation can alleviate drought and improve plant photosynthesis and growth (Bai et al., 2004; Farfan‐Vignolo & Asard, 2012), decreased precipitation will inhibit plant growth in this region (Ramírez et al., 2014; Xu, Zhou, & Shimizu, 2009). Though researchers have reported how warming and altered precipitation affect plant growth, these mechanisms do not always work. For example, in a semiarid grassland, Mueller et al. (2016) reported that warming initially decreased plant biomass but changed neutral later. Coincidentally, another experiment conducted in Mediterranean shrubland found that drought did not decrease plant biomass even in dry season (Sardans, Peñuelas, Prieto, & Estiarte, 2008).

One possible mechanism that explains unusual warming and altered precipitation effects may be that plant–plant competition buffers or obscures the effects of climate change on plant growth (Anke, Juergen, Jegor, & Carl, 2009; Ariza & Tielbörger, 2011; Liancourt et al., 2013). In a 5‐year field experiment in a northern California grassland, Suttle, Thomsen, and Power (2007) reported that the production of forbs increased in the first year under extended spring rainfall, but declined in the last two experimental years due to the positive responses of grass competitors to extended spring rainfall from the second year. In another field study in the southeastern Tibetan Plateau, Wang et al. (2016) found that warming increased tree density and growth over the short‐term, but this positive effect diminished because of spatial segregation resulting from competition‐induced thinning over time. Competitive status of plant species may determine their responses to climate change. For example, Tullus et al. (2017) observed that trees with high competitivity could benefit from elevated humidity and exhibited larger stem volume, but those with low competitivity had no responses to elevated humidity.

Plant tolerance to environmental stress under warming and altered precipitation could be another trait to influence plant growth. Plants with low tolerance to environmental stresses will be impacted first, and then, other plants may benefit from this variation, leading to some unpredictable responses to environmental changes. For example, heat stress induced by climate warming may reduce crop yields (Ortiz et al., 2008). However, heat‐tolerant variety may maintain or even increase yields under warming due to high photosynthetic rates (Bita & Gerats, 2013). Some plant species or varieties can enhance drought tolerance via changing their phenotypes to respond to drought differently (Olmo, Lopez‐Iglesias, & Villar, 2014). Thus, plant tolerance to environmental stresses should be considered together with plant–plant competition when predicting community dynamics under climate change scenarios. To our knowledge, few have examined these knowledge gaps from the perspectives of plant competition and tolerance to environmental stresses.

We conducted a warming and precipitation manipulation experiment in a semiarid grassland on the Loess Plateau to investigate the potential mechanisms that regulate plant community responses to climate change factors. A supplementary removal experiment was also carried out to characterize plant interactions in an attempt to understand how plant interactions may influence the effects of warming and altered precipitation on the plant community. We hypothesize that resource availability (namely water in this study) determines the primary mechanism(s) that controls community responses to climate change factors: Plant–plant competition dominates when resources are abundant, but plant tolerance to environmental stresses takes over when resources are limited.

## 2 MATERIALS AND METHODS

### 2.1 The study site and vegetation

The study site was located at Yunwushan Nature Reserve (106°21′–106°27′E, 36°10′–36°17′N, altitude 1,800–2,000 m, 6,700 hm^2^) on the Loess Plateau, Guyuan, Ningxia Hui Autonomous Region, China. The average annual temperature in this area is 7.01°C, with the highest mean monthly temperature of 22–25°C (July) and the lowest mean monthly temperature of −14°C (January). Mean annual precipitation is 425 mm, 60%–75% of which happens in July–September. Mean annual potential evaporation in this area is 1,330–1,640 mm. In the reserve, a dry steppe landscape established after more than thirty years’ enclosure and exclusion from grazing. This area is the largest region of typical steppe on the Loess Plateau in China, and the vegetation is representative of the native plant community. The vegetation is dominated by two perennial grasses and a subshrub. The dominant species are *Stipa grandis P. Smirn*., *Stipa przewalskyi Roshev*., and *Artemisia sacrorum Ledeb*., which consist of more than 70% of the total aboveground biomass (personal observation). *Artemisia sacrorum Ledeb*. often exhibits cluster growth. The soil is of the mountain gray‐cinnamon type classified as a Calci‐Orthic Aridisol, equivalent to a Haplic Calcisol in the FAO/UNESCO system (Qiu et al., 2015; Wei et al., 2016).

### 2.2 Experimental design and treatments

**Figure 1:**
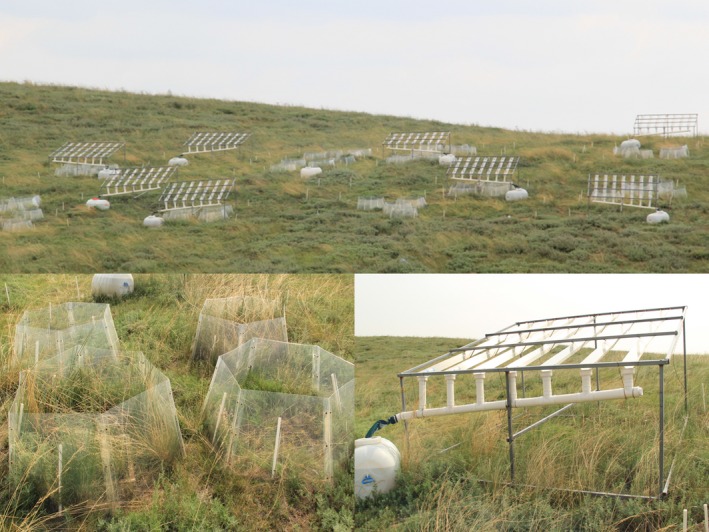
A partial overview of field experimental plots

A multifactor manipulation experiment was initiated in June 2015 on a mountaintop, where the topography is largely flat (Figure 1). There were three treatments in our experiment, including nitrogen addition (two levels: control and add 12 g N m^−2^ year^−1^), warming (two levels: ambient temperature and warming) and altered precipitation (three levels: precipitation addition (+30%), ambient precipitation, and precipitation reduction (−30%). In total, we had 12 treatments (2 nitrogen levels × 2 warming levels × 3 precipitation levels). Each treatment was replicated for 4 times, that is, 4 blocks, leading to a total of 48 plots. Each plot was 4 × 4 m in size and 1.5 m away from other plots in block. The distance between each block was 5 m. In this study, we just focus on the effects of warming and altered precipitation on plant communities, so data were only collected from 24 plots. The open‐top chamber (OTC) for the warming (W) treatment was the hexagonal OTCs made of transparent plexiglass, each with 1.19 m width at the top and 1.5 m at the bottom, 51.76 cm tall. (Figure 1). For the precipitation reduction (PR) treatment, multiple tilted v‐shaped transparent plexiglass was placed 1 meter above soil surface on a metal hanger over each plot (Figure 1). The transparent plexiglass covered 30% of the soil surface area and the precipitation blocked by the v‐shaped plexiglass was collected by plastic containers. The water collected in one plot was then manually added into the nearest plot that was designated for precipitation addition (PI) treatment within 24–48 hr after the rainfall event ended. In this way, each PI plot received an addition of 30% natural precipitation without changing the frequency of natural precipitation.

To supplement the result of the first experiment, a removal experiment was conducted in 2017 adjacent to the climate change experiment. There were three treatments in the removal experiment: nondominant species removed, the dominant subshrub removed, and both dominant grasses and nondominant species removed, in addition to an undisturbed control. A randomized block design was designed, leading to a total of 16 plots (0.5 m × 0.5 m in size for each plot). A walkway of 0.5 m was used designed between all the plots.

### 2.3 Plant sampling

To exclude the effects of stand litter on plant growth of next year, we mowed all the plants of each plot at 2 cm above soil surface in October 2015 when the branches and leaves had senesced. In August 2016, when plant community achieved peak biomass, we sampled aboveground plant biomass to estimate aboveground net primary productivity (ANPP). Plants in two 50 × 50 cm quadrats along a diagonal of each plot were cut from the soil surface. Plant samples in each quadrat were sorted by species, oven‐dried at 65°C for 48 hr, and weighed. The number of plant species occurred in the quadrats represented community species richness. In August 2017, we harvested aboveground plants biomass in a 50 × 50 cm quadrat on each plot of the removal experiment, dried, and weight. Finally, all data were converted into dry mass per square meter. We classified all plant species into nondominant species but the three dominant species. Richness and ANPP of nondominant species were the number of species and the sum of species ANPP in the collection, respectively. For the list of species, see Tables A1 and A2.

### 2.4 Soil characteristics

Soil temperature (10 cm depth) and soil moisture were measured once a week by portable temperature meter and TDR‐100 (SPectrum) in growth season of 2015 and 2016. In August 2016, soil samples were collected by taking 4 soil cores (2.5 cm dia) to 10 cm depth at each plot where plants were sampled. Soil pH was measured with a Mettler Toledo pH meter in a soil water suspension (1:2.5 wt/vol). A modified version (Fontaine et al., 2011) of the fumigation‐extraction method (Vance, Brookes, & Jenkinson, 1987) was used to measure microbial biomass carbon (MBC). Soil nitrate (NO_3_^−^) and ammonium (NH_4_^+^) contents were determined using a flow injection auto analyzer (SEAL‐AA3).

### 2.5 Data analysis

Data were divided into three groups for analyses: dominant grasses (DG), the dominant subshrub (DS), and nondominant species (ND). Two‐way ANOVAs with a block design were used to examine the main and interactive effects of warming and altered precipitation on soil temperature, soil moisture, ANPP, and species richness of community and groups. Because there were only one dominant subshrub and two dominant grass species and they almost occurred in all the plots, we did not test the effects of treatments on richness of dominant grasses and the dominant subshrub. Values of soil temperature and soil moisture were averaged by monthly values of the two experimental years. Data of species richness were ln‐transformed to meet normality assumptions of ANOVA. If the effect of any treatment or the interaction was significant on a parameter, we performed *Duncan* test to examine the differences between treatments. For the removal experiment, one‐way ANOVA was used to test the difference(s) of group ANPP with or without removing other group(s). All analyses were performed with SAS v.8.1 (SAS Institute Inc.).

Structural equation modeling (SEM) was conducted to examine hypothetical pathways that may explain how warming and altered precipitation impacted community ANPP and species richness. SEM could test interactive relationships between variables no matter they act as predictor and response variables (Grace, 2006; Veen, Olff, Duyts, & van der Putten, 2010). Five soil parameters, that is, ST, SM, soil pH, NO_3_^−^, and MBC, were initially included in the model. No effects of soil pH and MBC on aboveground plant biomass and species were detected so only ST, SM, and NO_3_ were included in the final model. Amos version 21.0.0 (Amos Development Corporation) with the maximum‐likelihood estimation method was used to parameterize the model. The *χ* ^2^ goodness‐of‐fit statistic and its associated *p* value were used to test the model fit to the data. A large *p* value associated with the *χ* ^2^ value, large GFI and CFI values, and small RMSEA value indicates that a model is good to predict relationships between variables.

## 3 RESULTS

### 3.1 Warming and altered precipitation experiment

#### 3.1.1 Soil temperature and moisture

**Figure 2:**
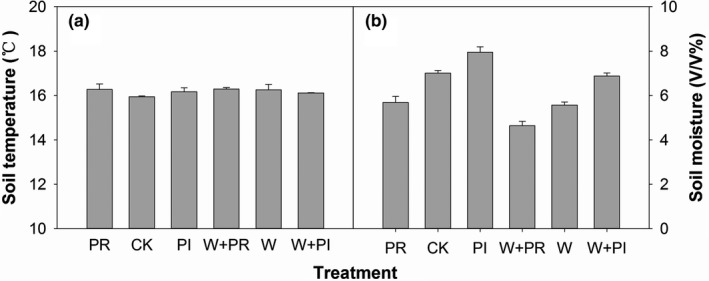
Effects of warming (W), precipitation reduction (PR), precipitation increase (PI) on soil temperature and moisture (10 cm under soil surface) across two years (2015–2016). The capital “CK” represents ambient

Warming, altered precipitation, and their combinations did not significantly affect soil temperature at 10 cm depth during 2015–2016 (Figure 2a; Table 1). In contrast, both warming and altered precipitation significantly impacted soil moisture (Figure 2b; Table 1). Warming decreased soil moisture by 17.09% in the experimental period (Figure 2b; Table 1). While precipitation addition increased soil moisture by 17.69%, precipitation reduction decreased soil moisture by 17.69% (Figure 2b; Table 1). There was no interaction effect of warming and altered precipitation on soil moisture (Table 1).

**Table 1:** Two‐way ANOVA of the effects of warming (W) and altered precipitation (P) and their interactions on soil temperature (ST) and soil moisture (SM)

Factor	ST		SM	
	*F*‐ratio	Pr > *F*	*F*‐ratio	Pr > *F*
W	0.36	0.5558	49.91	<0.0001
P	0.73	0.4980	59.66	<0.0001
W × P	0.85	0.4482	0.37	0.6975
Block	1.39	0.2841	0.38	0.7706

Note

The effects of block were also considered in data analysis. *F*‐ratios and *p* values were shown.

#### 3.1.2 Plant species richness and plant ANPP

**Figure 3:**
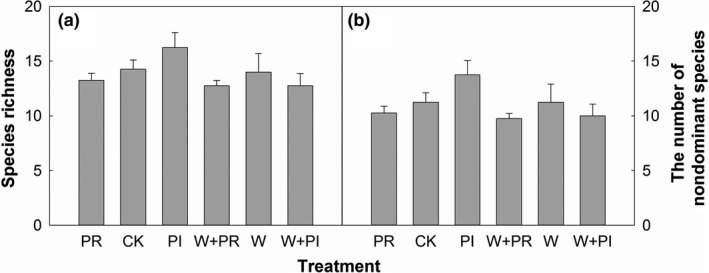
Effects of warming (W), precipitation reduction (PR), precipitation increase (PI) on community level species richness (a) and the number of nondominant species (b) in 2016

Warming marginally decreased species richness of the communities and ND species (Figure 3; Tables 2 and 3). Altered precipitation had no effects on species richness of either the communities or the ND species (Figure 3; Tables 2 and 3).

**Table 2:** Two‐way ANOVA of the effects of warming (W) and altered precipitation (P) and their interactions on community (T), dominant species (D), nondominant species (ND), dominant grasses (DG), and the dominant subshrub (DS) aboveground net primary productivity (ANPP) and species richness

	Treatment	T		D		ND		DG		DS	
		*F*	*p*	*F*	*p*	*F*	*p*	*F*	*p*	*F*	*p*
ANPP	W	0.74	0.4032	0.15	0.7050	4.63	0.0481	1.11	0.3089	0.24	0.6332
	P	3.08	0.0757	3.93	0.0426	6.16	0.0111	1.42	0.2728	3.08	0.0756
	W × P	1.17	0.3377	1.32	0.2958	0.35	0.7071	0.03	0.9659	1.61	0.2326
	Block	1.71	0.2087	1.12	0.3731	0.60	0.6241	0.6	0.6236	0.59	0.6295
Species richness	W	3.20	0.0938	–	–	3.67	0.0748	–	–	–	–
	P	0.99	0.3942	–	–	1.73	0.2108	–	–	–	–
	W × P	1.45	0.2661	–	–	2.04	0.1644	–	–	–	–
	Block	1.77	0.1967	–	–	2.45	0.1039	–	–	–	–

Note

The numbers of dominant species, dominant grasses, and the dominant subshrub were not analyzed because they were really small (three species in total) and changed little. The effects of block were also considered in data analysis. *F*‐ratios and *p* values were shown.

**Table 3:** Multiple comparisons of community (T), dominant species (D), nondominant species (ND), dominant grasses (DG), and the dominant subshrub (DS) ANPP and species richness used *Duncan Text* with *p* < 0.05

Factors	Treatment	ANPP					Species richness				
		T	D	ND	DG	DS	T	D	ND	DG	DS
Warming	CK	241.73a	163.28a	78.46a	53.80a	109.52a	14.58a	–	11.75a	–	–
	W	227.48a	170.99a	56.50b	71.49a	99.50a	13.17a	–	10.33a	–	–
Precipitation	CK	214.74b	127.88b	86.88a	52.05a	75.85b	14.13a	–	11.25a	–	–
	PI	262.95a	191.08a	71.89a	53.25a	137.85a	14.50a	–	11.88a	–	–
	PR	226.12ab	182.46a	43.68b	82.64a	99.83ab	13.00a	–	10.00a	–	–

Note

The same with Table 2, the numbers of dominant species, dominant grasses, and the dominant subshrub were not analyzed. Mean values were shown.

**Figure 4:**
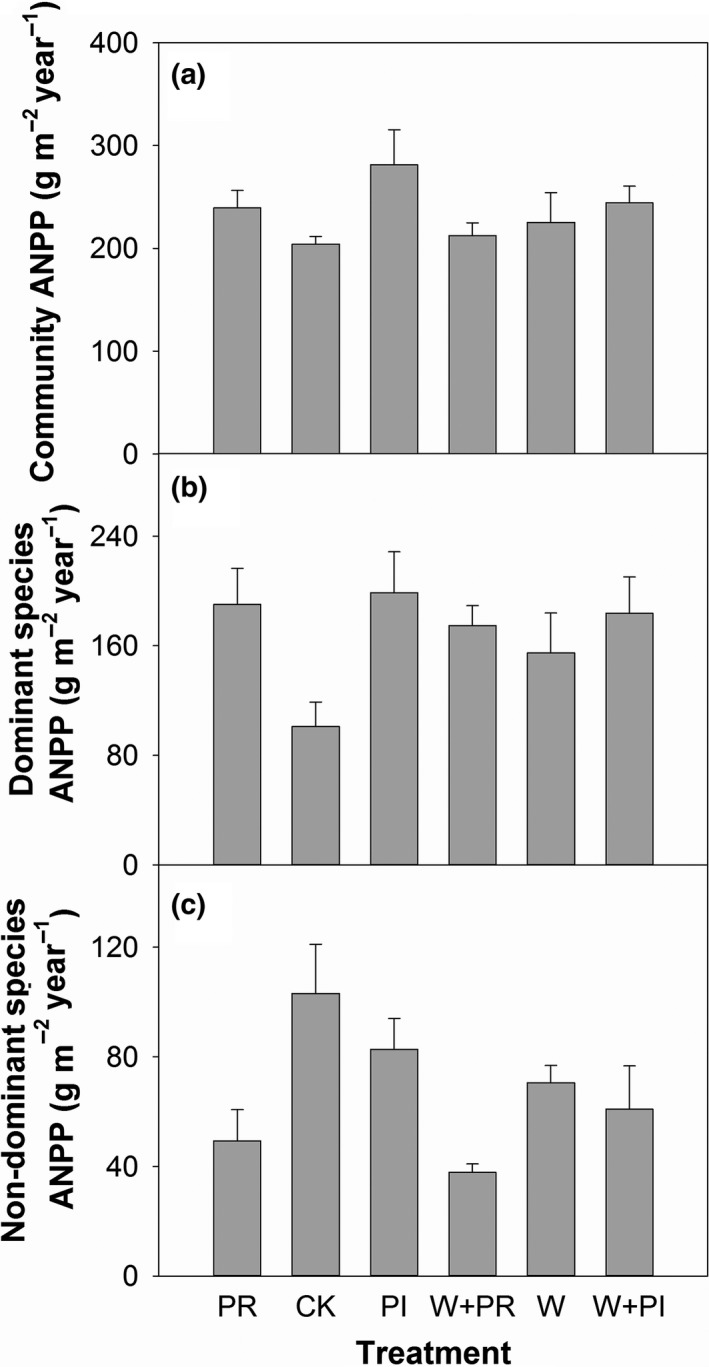
Effects of warming (W), precipitation reduction (PR), precipitation increase (PI) on community aboveground net primary productivity (ANPP) (a), dominant species ANPP (b), and nondominant species ANPP (c) in 2016

**Figure 5:**
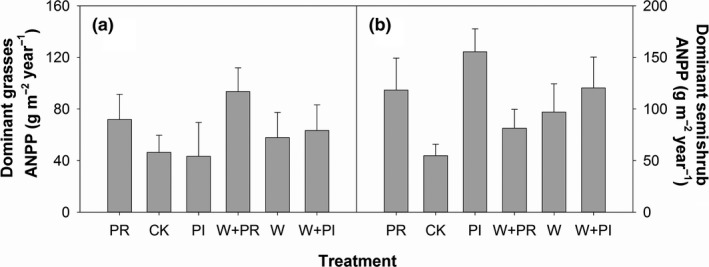
Effects of warming (W), precipitation reduction (PR), precipitation increase (PI) on dominant grasses ANPP (a) and the dominant subshrub ANPP (b) in 2016

Warming had no effects on community ANPP (Figure 4a; Table 2) and ANPP of DG (Figure 5a; Table 2) and DS (Figure 5b; Table 2), but significantly decreased ANPP of ND species (Figure 4c; Table 2). In contrast, precipitation addition significantly increased community ANPP (Figure 4a; Table 3). Precipitation reduction did not change community ANPP (Figure 4a; Table 3). Altered precipitation, both added and reduced precipitation, increased dominant species ANPP (Figure 4b; Table 3). Precipitation addition increased DS ANPP, and precipitation reduction had no effect on DS ANPP (Figure 4a,b; Tables 2 and 3). Altered precipitation did not affect DG ANPP (Figure 4a,b; Tables 2 and 3). Precipitation reduction significantly decreased ND ANPP (Figure 4c; Table 3). No interactive effects of warming and altered precipitation on plant ANPP were detected in any plant species groups (all *p* > 0.05).

#### 3.1.3 Structure equation model

**Figure 6:**
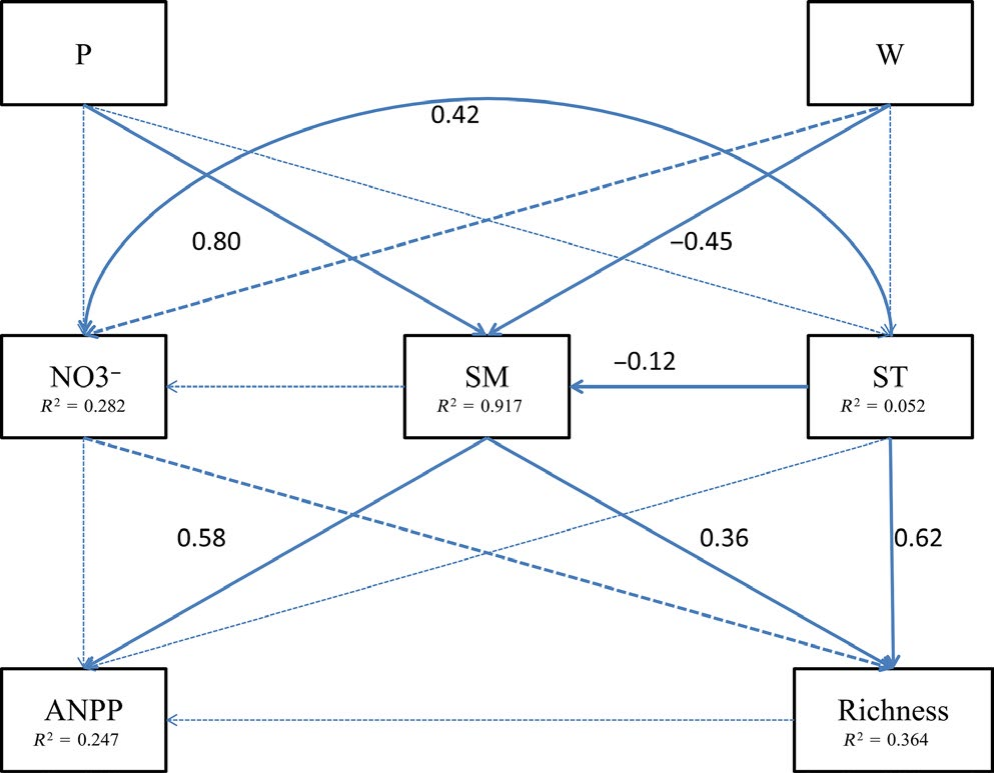
Final model results of structural equation modeling (SEM) analysis for warming and altered precipitation on community level ANPP and species richness via pathways of soil nitrate content, soil moisture, and soil temperature. Square boxes represent variables included in the models. Results of model fitting: (*χ* ^2^ = 3.959, *p* = 0.555, *df* = 5, GFI = 0.957, CFI = 1.000, RMSEA = 0.000). Solid arrows the directions and significant effects (*p* < 0.05); thick dashed arrows denote the directions and Marginal significant effects (*p* < 0.1); fine dashed arrows denote the directions and with no significant effects (*p* > 0.1). Values beside the solid arrows represent standardized path coefficients. Abbreviations: NO_3_^−^, soil nitrate content; P, altered precipitation; SM, soil moisture; ST, soil temperature; W, warming

Results of structure equation model showed that warming and altered precipitation impacted plants by modifying soil moisture but not temperature (Figure 6). Soil moisture was positively related to precipitation and negatively related to warming (Figure 6). There was a positive relationship between soil moisture and plant species richness and ANPP (Figure 6). While soil temperature was not significantly related to plant ANPP, it was positively related to plant species richness (Figure 6).

### 3.2 Removal experiment

**Figure 7:**
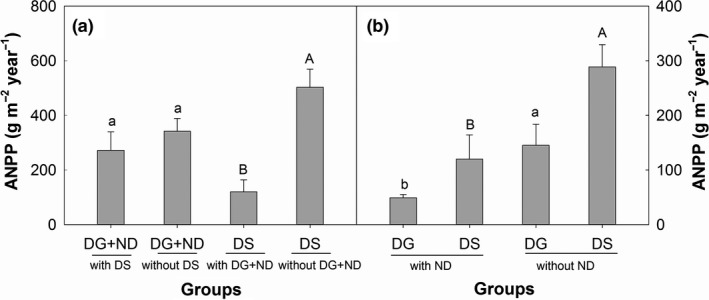
Effects of removing group(s) on ANPP of other group(s). (a) the effects of removing DS on ANPP of DG plus ND and removing both DG and ND on ANPP of DS. (b) the effects of removing ND on ANPP of DG and DS. The different lowercase or uppercase letters on the bars denoted significant differences between remove of not remove group(s) treatments (*p* < 0.05)

Removal of the dominant subshrub (DS) did not significantly affect ANPP of dominant grasses (DG) plus nondominant species (ND). In contrast, removal of DG and ND significantly increased DS ANPP by 319.05% (Figure 7a; Table 4). Removal of nondominant species significantly increased DG and DS ANPP by 195.01% and 140.45%, respectively (Figure 7b; Table 4).

**Table 4:** One‐way ANOVA of the effects of removing group(s) on ANPP of other group(s)

Treatment	Group	*F*‐ratio	Pr > *F*
Remove DS	ND + DG	0.73	0.4257
Remove ND	DG	6.08	0.0487
Remove ND	DS	7.88	0.0309
Remove ND + DG	DS	23.16	0.0030

Note

*F*‐ratios and *p* values were shown.

## 4 DISCUSSION

### 4.1 Aboveground net primary productivity responds to warming and altered precipitation

Ecosystem productivity is considered closely related to species richness (Willig, 2011). However, in our study, warming and altered precipitation did not affect plant ANPP via species richness because of nonsignificant effects of warming and altered precipitation on species richness (Figure 3a) and the poor relationship between species richness and ANPP (Figure 6).

Warming could impact plant growth directly via changing photosynthesis (Albert et al., 2011; Tang et al., 2007) and indirectly by altering water availability and microbial nutrient release (De Boeck et al., 2006; Lin, Xia, & Wan, 2010). However, though warming negatively affected soil moisture (Figure 6), warming did not significantly decreased community ANPP in our study. This contrasts with the results of many other studies in which warming either increased (Collins et al., [Link]; Cowles, Wragg, Wright, Powers, & Tilman, 2016; Wu, Dijkstra, Koch, Peñuelas, & Hungate, 2011) or decreased (Rustad et al., 2001) aboveground plant biomass. Similar to the warming effect, precipitation reduction also decreased soil moisture but did not change community ANPP, which was again different from results from most previous studies (Hoover, Knapp, & Smith, 2014; Reichmann & Peters, 2013). With the knowledge of precipitation is the most crucial factor to affect plant growth in arid regions (Knapp et al., 2008; Zeppel, Wilks, & Lewis, 2014), the results suggest that our warming and precipitation reduction treatments provided moderate drought (Fraser et al., 2013) and did not threaten plant survival at the community level. Another possibility is that with 30 years of recovery, the plant community at our site had developed large root systems (Bai, Su, & Cheng, 2013) to resist moderate drought. Contrasting with warming and precipitation reduction, precipitation addition enhanced soil water availability to improve root activity and leaf photosynthesis (Fay, Kaufman, Nippert, Carlisle, & Harper, 2008) and then increase plant ANPP.

Though numerous studies reported the interactive effects of warming and altered precipitation on plant biomass (Hoeppner & Dukes, 2012; Luo, Gerten, & Maire, 2008), our results showed no interactions between warming and altered precipitation on affecting plant. Winkler, Chapin, and Kueppers (2016) argued that available soil moisture largely determined the responses of a forb‐dominated alpine community to warming, in which simulated warming negatively affected aboveground biomass at the community level by decreasing available soil moisture but had no effect when soil moisture was adequate. In our study, however, soil moisture was deficient all the time even in water addition plots. Therefore, plant growth was closely related to soil water rather than temperature in our water‐limited ecosystem. Both warming and precipitation alteration affected plants by altering soil water availability to plants and did not interactively impact plant biomass and species richness.

### 4.2 Competition and tolerance to drought modulate the responses of plant growth to warming and altered precipitation

In semiarid grasslands on Loess Plateau, it was reported that changing soil water availability could significantly impact plant ANPP (Zhao, Wu, Gao, Tian, & Li, 2014). Thus, precipitation addition can improve soil water availability and increase plant ANPP. What was interesting is that precipitation addition only increased the ANPP of the dominant subshrub but not dominant grasses or nondominant species (Figure 4a–c). Robertson, Zak, and Tissue (2010) argued that large magnitude of precipitation addition was needed to affect all plant groups. However, the magnitude of precipitation addition could not be the reason in this study because precipitation addition had no trend to increase both ANPP of DG (Figure 5a; Table 3) and ND (Figure 4c; Table 3). Different species often interact each other in a community, which can modify the effects of climate changes on plant communities (Suttle et al., 2007; Tullus et al., 2017). Thus, varied responses of plant groups to precipitation addition may be due to species interactions (Suttle et al., 2007). Our removal experiment showed that the dominant subshrub competed resources with dominant grasses and nondominant species (Figure 7a; Table 4). This competition relationship modified the effect of precipitation addition on plant community. The competitive capacity of the dominant subshrub is stronger than dominant grasses and nondominant species because of clonal growth pattern and allelopathy (Nilsson, 1994; Wang, Xie, & Yang, 2011). As a result, the dominant subshrub benefited the most in precipitation addition plots and grown more biomass.

Besides competition, tolerance to stress may also decide plant survival in extreme environment. Ambient soil moisture in our site was about 7% v/v (Figure 1b), which was lower than that of many other semiarid grasslands (higher than 9% v/v in average) (Kurc & Small, 2007; Yang et al., 2011), indicated that drought stress was more severe in our study site. The result of ANPP of dominant grasses plus nondominant species did not increased when removed the dominant subshrub (Figure 7a) also showed the severe drought stress. Warming and precipitation reduction decreased soil moisture by 17.69% and 17.69% (Figure 2b), respectively, which aggravated the water limitation of plant community. In this condition, drought stress may threaten plant growth.

In a previous study, Zavaleta, Shaw, Chiariello, Mooney, and Field (2003) reported that global changes affected plant species richness primarily driven by changes in forbs richness. Collins et al. ([Link]) found that warming significantly increased biomass of forbs after a wildfire. These results, together with our findings, suggested that forbs were likely to be highly sensitive to environmental changes and were decreased primarily by drought. The decline of nondominant species provided more space and/or resources for dominant species to grow, thereby compensating the biomass loss due to depression of nondominant species. Additionally, dominant grasses benefited more from the decline of nondominant species than the dominant subshrub (Figures 4a,b and 7b) probably because of their habitat overlaps with nondominant species at our study site.

## 5 CONCLUSIONS

Plant interactions may critically affect the responses of communities to climate change. We conducted two field experiments to assess the effect of plant interactions on community responses to warming and altered precipitation. Our results indicate that interspecific competition modulated the effects of warming and altered precipitation on plant community when resources were less limited and plant tolerance to drought took over when resources were more limited. These results suggest that responses to climate changes at the ecosystem or community level may be less variable than those at the plant species level when different species or functional groups compete for the same limited resources. However, such mechanism may be untenable in plant communities in which species are mutually beneficial.

## CONFLICT OF INTEREST

None declared.

## AUTHORS' CONTRIBUTIONS

S.J.H., H.G., and Y.W. designed the experiment. F.L.S, Y.N.W., J.X.G., J.J.Z., and F.W.W. carried out the plant and soil samples analysis. F.L.S., S.J.H., and H.G. interpreted the results, and wrote and edited the manuscript. All authors contributed to the manuscript writing and gave final approval for publication.

## Data Availability

Soil characteristics and aboveground plant biomass data are available from the Dryad Digital Repository (https://doi.org/10.5061/dryad.3b71520).

## APPENDIX 1

**Table A1:** Plant species appeared in our experiment plots

Species Name	Family	Group
*Adenophora stricta* Miq.	Campanulaceae	ND
*Agropyron cristatum* (L.) Gaertn.	Gramineae	ND
*Allium tenuissimum *L.	Liliaceae	ND
*Androsace mariae* Kanitz	Primulaceae	ND
*Artemisia frigida* Willd.	Asteraceae	ND
*Artemisia gmelinii*	Asteraceae	DS
*Artemisia pubescens Ledeb*.	Asteraceae	ND
*Astragalus scaberrimus Bunge*	Leguminosae	ND
*Carduus nutans *L.	Asteraceae	ND
*Carex aridula*	Cyperaceae	ND
*Dendranthema lavandulifolium (Fisch. ex Trautv.) Ling & Shih*	Asteraceae	ND
*Cleistogenes squarrosa (Trin*.*) Keng*	Gramineae	ND
*Delphinium grandiflorum *L.	Ranunculaceae	ND
*Dracocephalum heterophyllum Benth*.	Labiatae	ND
*Galium verum Linn*.	Rubiaceae	ND
*Gentiana macrophylla Pall*.	Gentianaceae	ND
*Heteropappus altaicus (Willd*.*) Novopokr*.	Asteraceae	ND
*Koeleria cristata (*L.*) Pers*.	Gramineae	ND
*Leontopodium leontopodioides (Willd*.*) Beauv*.	Asteraceae	ND
*Leymus secalinus (Georgi) Tzvel*.	Gramineae	ND
*Medicago Sativa Linn*.	Leguminosae	ND
*Potentilla acaulis* L.	Rosaceae	ND
*Potentilla bifurca*	Rosaceae	ND
*Potentilla tanacetifolia Willd*.* ex Schlecht*.	Rosaceae	ND
*Bupleurum scorzonerifolium Willd*.	Umbelliferae	ND
*Salsola collina Pall*.	Chenopodiaceae	ND
*Saussurea alata DC*.	Asteraceae	ND
*Scutellaria scordifolia Fisch. ex Schrank var. villosissima C.Y.Wu & W.T.Wang*	Labiatae	ND
*Stellera chamaejasme Linn*.	Thymelaeaceae	ND
*Stipa grandis P. Smirn*.	Gramineae	DG
*Stipa przewalskyi Roshev*.	Gramineae	DG
*Taraxacum mongolicum Hand.‐Mazz*.	Asteraceae	ND
*Thalictrum petaloideum *L.	Ranunculaceae	ND
*Thesium refractum C. A. Mey*.	Santalaceae	ND
*Thymus mongolicus Ronn*.	Labiatae	ND
*Torularia humilis (C. A. Mey.) O. E. Schulz*	Brassicaceae	ND
*Viola dissecta*	Violaceae	ND
*Sp1*		ND

Abbreviations: DS, the dominant subshrub; DG, dominant grasses; ND, nondominant species.

**Table A2:** Species richness in plant removal plots

Plots	Treatment	Species richness
1	FG	7
6	FG	6
12	FG	5
15	FG	11
2	FGS	5
7	FGS	6
9	FGS	9
16	FGS	14
3	GS	3
8	GS	3
10	GS	3
13	GS	3
4	S	1
5	S	1
11	S	1
14	S	1

Abbreviations: FG, remove the dominant subshrub; FGS, undisturbed control; GS, remove nondominant species; S, remove both dominant grasses and nondominant species.
